# Age dependent susceptibility and immune responses to La Crosse virus infection in non-human primates

**DOI:** 10.1038/s41598-025-01285-8

**Published:** 2025-05-13

**Authors:** Clayton W. Winkler, Tyson A. Woods, Aaron B. Carmody, Katherine G. Taylor, Rachel LaCasse, Dana Scott, Patrick W. Hanley, Jamie Lovaglio, Karin E. Peterson

**Affiliations:** 1 Laboratory of Neurological Infections and Immunity, Department of Intramural Research, National Institute of Allergy and Infectious Diseases, Rocky Mountain Laboratories, National Institutes of Health, 903 S. 4th St., Hamilton, MT 59840 USA; 2 Research Technologies Branch, Rocky Mountain Laboratories, Department of Intramural Research, National Institute of Allergy and Infectious Diseases, National Institutes of Health, Hamilton, MT USA; 3 Rocky Mountain Veterinary Branch, Rocky Mountain Laboratories, Department of Intramural Research, National Institute of Allergy and Infectious Diseases, National Institutes of Health, Hamilton, MT USA

**Keywords:** La Crosse virus, Non-human primate, Age-related susceptibility, Immune response, Interferon stimulated gene, T cell, Viral host response, Lymphocyte activation, Viral infection, Dendritic cells, Viral pathogenesis

## Abstract

La Crosse virus (LACV) is a primary cause of pediatric viral encephalitis in the United States but rarely causes disease in adults. We tested whether cynomolgus macaques displayed a similar age-dependent susceptibility to LACV. Immune responses from naïve or LACV infected weanling (9–15 months), juvenile (19–23 months) or adult (> 6 years) animals were measured and infected animals were monitored for disease. Naïve weanling animals had fewer dendritic cells in their blood and weaker induction of IFN-stimulated genes (ISG) and chemokines when PBMCs were stimulated in vitro. While no infected animals developed disease, the weaker innate response in naive weanlings correlated with increased viral RNA in plasma from 2 of 3 infected weanlings out to 7 days post infection (dpi). Activated CD8^+^ T cells and neutralizing antibody proportions were similar amongst all ages. However, CD4^+^ T cells proportions were increased in young animals relative to adults. This suggests the CD4^+^ adaptive response in young animals may be bolstering an initially weak innate response to clear virus. Finally, because macaques were resistant to disease, we infected 3 common marmosets intranasally with LACV. Marmoset were selected due to their susceptibility to viral encephalitis. Although no animals showed disease signs, one animal had evidence of infection in the nasal mucosa out to 23 days with associated vacuolization, edema and immune cell infiltration.

**Supplementary Information:**

The online version contains supplementary material available at 10.1038/s41598-025-01285-8.

## Introduction

La Crosse Virus (LACV) is a single stranded, negative-sense RNA virus belonging to the California serogroup of Orthobunyaviruses, that can infect humans and cause neurological disease^1^. LACV is transmitted primarily by the eastern tree hole mosquito, *Aedes triseriatus*, which has a range that includes the eastern US and southeastern Canada^2^. Recently surveys have found that the range of *Aedes triseriatus* is expanding in Canada possibly due to climate change^3^. LACV has also recently been detected in two other species, *Aedes albopictus* and *Aedes japonicus*^4^, which have a broader range across North America than the canonical host. Thus, due to the increasing vectors and vector ranges, the incidence of cases of LACV in humans could increase.

The majority of LACV infections of humans are asymptomatic or induce a mild febrile illness^5^. However, some LACV infections can result in severe encephalitis, with clinical outcomes ranging from behavioral changes, cognitive defects and seizures to coma or death^6^. Interestingly, over 90% of the reported neurological disease cases caused by LACV occur in children under 16^7^, with the primary age of patients ranging between 4 and 11 years of age^8^. The susceptibility of children, but not adults, to LACV-encephalitis suggests that age-related host factors influence susceptibility to LACV encephalitis.

Studies by our lab and others have shown a similar age-related susceptibility to LACV encephalitis in mice. Peripheral infection of LACV through subcutaneous (s.c.), intraperitoneal (i.p.) or intradermal (i.d.) inoculation results in severe neurological disease in mice three weeks of age or younger^9,10^, but not in mice over six weeks of age^11,12^. Differences in the type I interferon (IFN) response to LACV infection contribute to this difference in susceptibility with higher type I IFN responses by myeloid dendritic cells to LACV infection in adults suppressing viremia, while low type I IFN responses in young mice fail to control virus replication^11^. Inhibition of the type I IFN response in adult mice results in increased incidence of encephalitis, while treatment with type I IFN blocks disease in young mice^11^. In another study, constitutive expression of the human IFN-stimulated gene (ISG) MxA in *Ifnar1*^−/−^ adult mice partially rescues age-related resistance^13^. Thus, the type I IFN response has an important role in age-dependent susceptibility in mice.

In addition to the type I IFN response, the adaptive immune response also influences age-related susceptibility to LACV encephalitis in mice. Although depletion of T cells and/or B cells does not affect disease kinetics in young mice, depletion of T cell and B cells responses in adult mice increases incidence of neurological disease^14^. Similarly, passive transfer of adult, neutralizing antibody-contain serum, and adoptive transfer of splenocytes from immune adult mice to naïve weanling mice increases survival following LACV infection^15^. Thus, components of both the innate and adaptive immune response appear to contribute to protection against LACV in mice.

Despite the clinical significance of LACV in humans, few attempts have been made to establish non-human primate (NHP) models of infection and disease to test potential therapeutics, vaccines or study NHP immune response to the virus. One study inoculated rhesus macaques, *Macaca mulatta*, with LACV via the intramuscularly or subcutaneously routes^9^. These animals do not develop clinical disease. They do, however, mount an immune response to virus, even at a low 10^1^ plaque forming unit (PFU) dose, as indicated by elevated neutralizing antibody titers and blood chemistries suggesting the animals were productively infected. A second study infected juvenile (2–3 years old) rhesus macaques intracerebrally with LACV and found the virus infected neurons but was cleared from the brain by 14 days after infection^16^. Innate and adaptive immune responses were detected in the brain up to 21 dpi, but no animals developed disease. Importantly, no viremia was detected in infected animals which calls into question whether virus is present in the periphery which could be an important site of replication^17^ to seed further brain infection. Regardless, whether there is an age difference in susceptibility to viral infection or in the immune responses to LACV in NHPs has not been examined. Additionally, NHP genera and species other than rhesus macaque may be more susceptible to LACV infection. For example, the common marmoset, *Callithrix jacchus*, are naturally susceptible to multiple encephalitic viruses and might be a useful model for LACV^18,19^.

To determine how age impacts LACV infection and immune response in a NHP model, we infected cynomolgus macaques, *Macaca fascicularis*, at three different ages: weanling (≤ 15 months), juvenile (19–23 months) and adult (> 6 years). Cynomolgus macaques were selected due to both being immediately available and a species in which LACV infection has not yet been examined. Although no animal developed neurological disease, we found age-related differences in viremia as well as innate and adaptive immune responses. Additionally, we examined whether common marmosets were susceptible to LACV encephalitis. While all animals were asymptomatic, infection, pathology and immune cell recruitment in the nasal mucosa were evident.

## Results

### Naïve innate immune response differs between young and older macaques

**Fig. 1:**
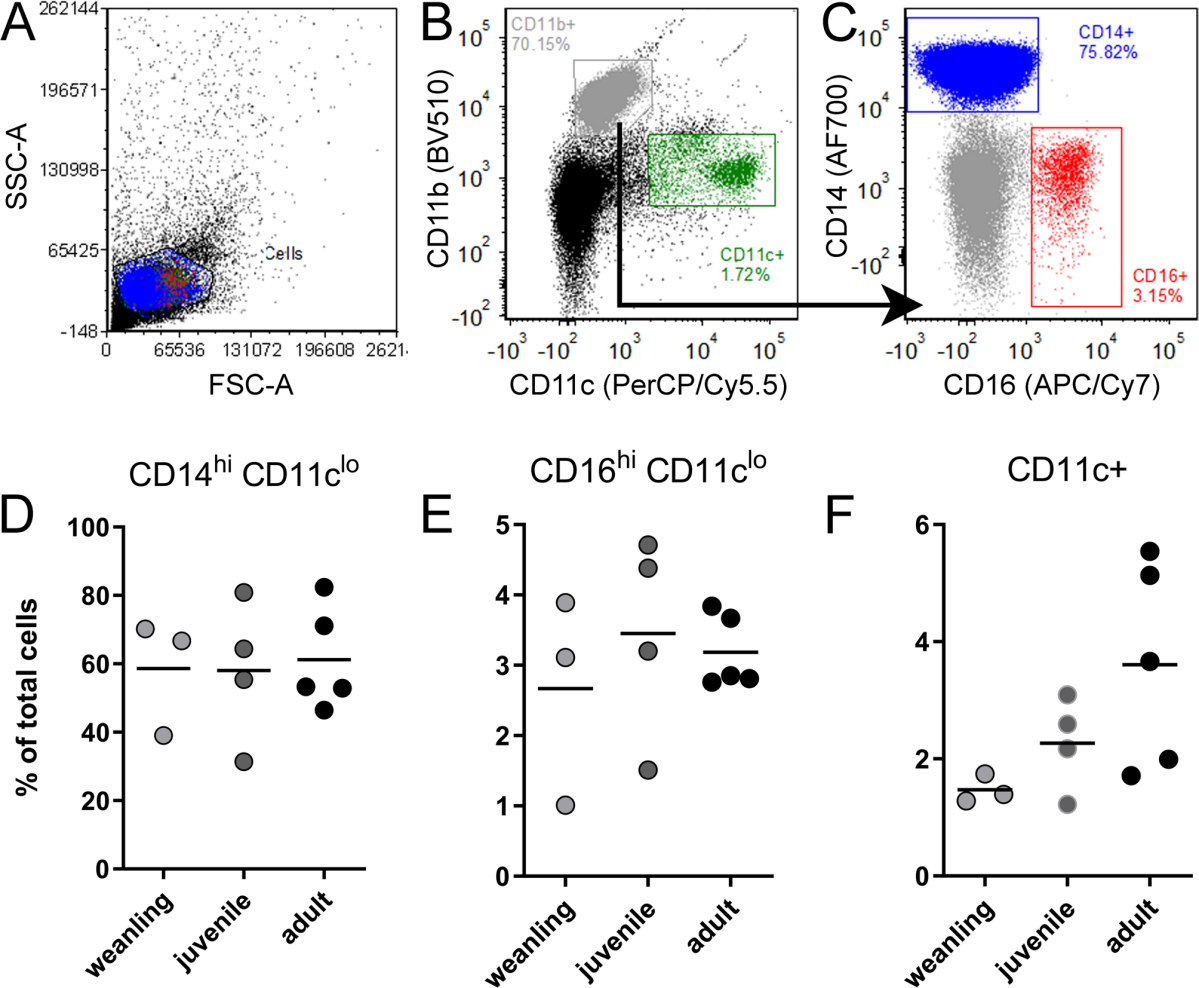
Dendritic cells proportions of are lower in naïve weanling cynomolgus macaques relative to older animals. Flow cytometric analysis of PBMCs from naïve weanling, juvenile and adult cynomolgus macaques was performed to identify CD11b^+^ CD14^+^ CD11c- monocytes (**A**-**C**), CD11b^+^ CD16^+^ CD11c- monocytes (**A**-**C**) and CD11c^+^ dendritic cells (**A**,**B**). Quantifications of the proportions of each cell type as a proportion of total PBMCs is in shown in (**D**-**F**).

Due to the importance of the innate immune response and myeloid DCs in controlling LACV infection in mice^11^, we first analyzed the proportions of monocytes and DCs in peripheral blood mononuclear cells (PBMCs) of naïve cynomolgus macaques at different ages. We compared three age groups: <15 months (weanling), 19–23 months (juvenile) and > 6 years of age or older (adult). Analysis of PBMCs showed no difference in the age groups in the relative proportions of CD14^hi^ CD11c^lo^ and CD16^hi^ CD11c^lo^ monocytes (Fig. 1A-E). While the overall proportions of CD11c^+^ DCs were also not statistically different, weanling animals tended to have lower proportions of these cells compared to the other groups (Fig. 1C), which was reminiscent of our findings in weanling mice.

**Fig. 2:**
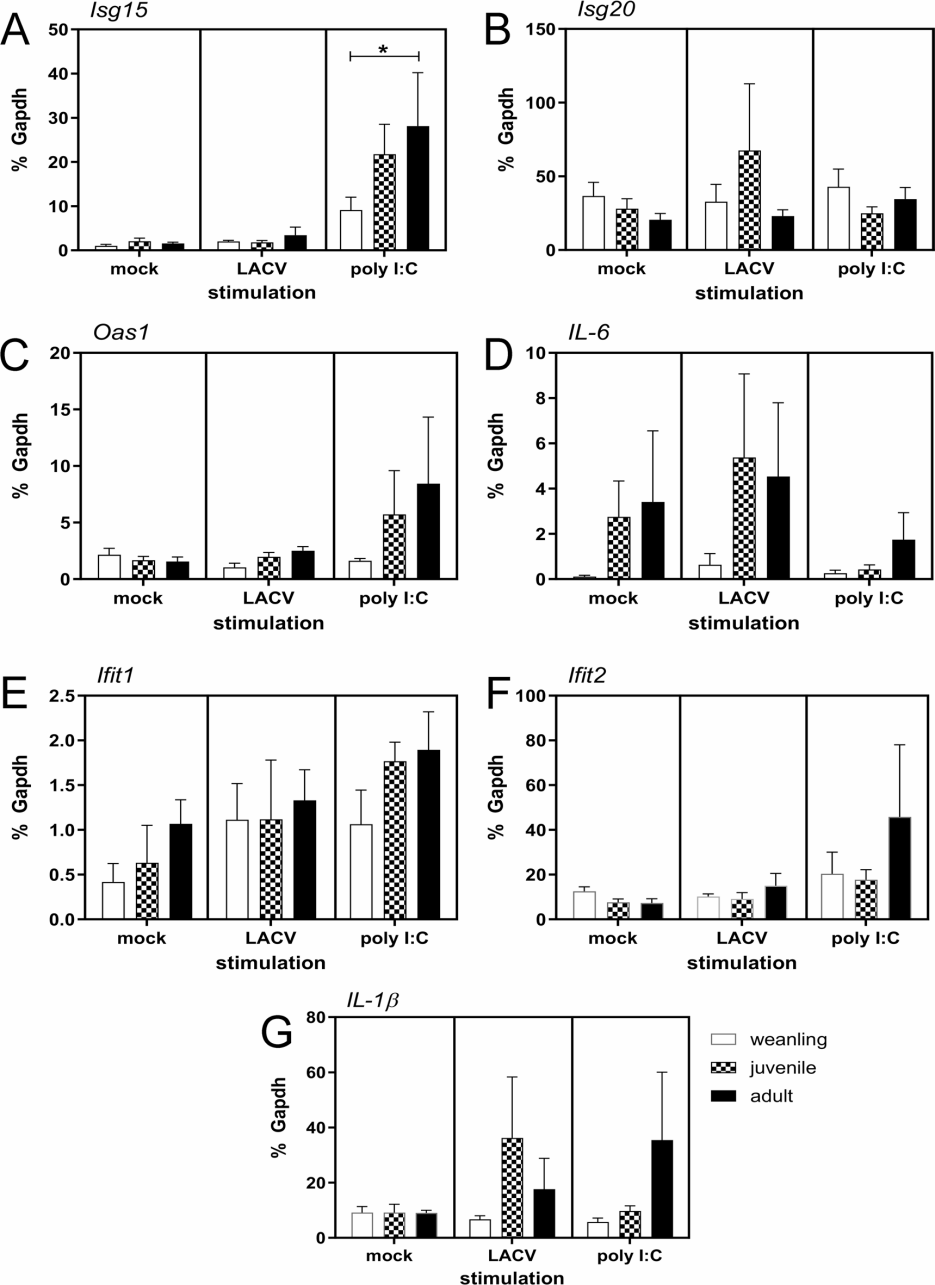
Naïve weanling cynomolgus macaques have a weaker early innate response to virus and synthetic viral RNA mimics. PBMCs isolated from naïve weanling, juvenile and adult cynomolgus macaques were cultured for 5 h with mock inoculum, LACV (MOI 0.01) or the viral double-stranded RNA mimic poly I: C (1 µg/ml). RNA was isolated from cells, cDNA synthesized and qRT PCR assays for *Isg15* (**A**), *Isg20* (**B**), *Oas1* (**C**), *Il6* (**D**), *Ifit1* (**E**), *Ifit2* (**F**) and *Il-1b* (**G**) transcripts were performed. Target transcript expression is plotted as a percent of the house-keeping transcript *Gapdh*. A two-way ANOVA with a Dunnett’s multiple comparisons test was used to compare the indicated transcript expression between each of the three age groups. *p < 0.05.

To examine whether PBMCs from weanling macaques had reduced IFN-stimulated gene (ISG) responses compared to juvenile or adult animals, PBMCs were isolated and stimulated in vitro with either LACV or poly I: C and analyzed for ISG and cytokine transcript expression using real-time PCR. Although expression levels were variable between animals, there were differences between age groups in ISG and cytokine gene expression (Fig. 2). PBMCs from adults typically generated the strongest responses to either stimulation, except for LACV-induced *IL-1β* and *Isg20* where juveniles had the strongest response. Conversely, PBMCs from weanling animals had consistently weaker responses to either LACV or poly I: C stimulation. The smaller ISG and cytokine response in PBMCs from weanlings compared to PBMCs from juveniles or adults correlate with the lower percentage of DCs in the blood at this age (Fig. 1C). Thus, weanling animals may not be able to mount as strong of an innate immune response as older animals to virus infection.

### Impact of age on LACV infection and neurological disease

To examine whether age impacts LACV infection in macaques, we infected three animals in each age group by subcutaneous (sc) inoculation with 10^7^ PFU of LACV (Table 1). One juvenile (19 months) and one adult (> 8 years) were used as uninfected controls. Animals were evaluated daily for any signs of neurological disease using a ranked scoring system. Additionally, blood was drawn at 3, 7, 14, and 21 days post infection (dpi). Analysis of plasma by plaque assay did not show detectable levels of virus (Table 1). However, real-time PCR analysis for viral RNA showed positive plasma samples for one weanling and one juvenile at 3 dpi and one weanling at 7 dpi was strongly positive and a second weanling was faintly positive (Table 1). Thus, some young animals, but none of the adults, had detectable viral transcripts in plasma at early timepoints post-infection.

**Table 1:** Cynomolgus macaques.

	ID^a^	Months	Sex	Inoculum	Viral RNA in plasma, and plaque forming units^b^				Neutralizing antibody^c^	Clinical signs	IHC
					3 dpi	7 dpi	14 dpi	23 dpi	23-28dpi		
Weanlings	WL-1	9	M	LACV	++, np	+, np	-, np	-, np	2048	-	-
	WL-2	12	F	LACV	-, np	-, np	-, np	nt, nt	2048	-	nt
	WL-3	15	F	LACV	-, np	++, np	-, np	nt, nt	512	-	nt
Juveniles	JM-1	19	F	PBS	-, np	-, np	-, np	nt, nt	-	-	nt
	JL-1	20	F	LACV	++, np	-, np	-, np	-, np	512	-	-
	JL-2	23	F	LACV	-, np	-, np	-, np	nt, nt	2048	-	nt
	JL-3	23	M	LACV	-, np	-, np	-, np	nt, nt	2048	-	nt
Adults	AM-1	103	F	PBS	-, np	-, np	-, np	nt, nt	-	-	nt
	AL-1	75	M	LACV	-, np	-, np	-, np	nt, nt	512	-	nt
	AL-2	100	F	LACV	-, np	-, np	-, np	-, np	512	-	-
	AL-3	103	F	LACV	-, np	-, np	-, np	nt, nt	2048	-	nt

^a^: W = weanling, J = juvenile, A = adult, M = mock and L = LACV. ^b^: ++: 6–9 of 9 wells positive (detects down to 10 PFU); +: 2–5 wells positive; - : 0–1 well positive. np = no plaques detected in plaque assay. *nt* = not tested. ^b^: dilution of plasma that neutralized ≥ 50% of virus plaque formation of a known virus stock. – indicates no clinical signs, neutralizing antibody or histopathological findings. *nt* not tested.

To determine if detectable viral RNA in plasma was associated with virus infection or pathology in tissues, we histologically examined tissues, including brain, from weanling LACV (WL)-1, juvenile LACV (JL)-1 and adult LACV (AL)-2 at 23 dpi. The weanling and juvenile animals were selected because they had detectable viral transcripts at 3 dpi (Table 1) and the adult was taken as a sample for that age group. No virus was detected by immunohistochemistry (IHC) in any animal and no virus-related histopathological findings were observed in any tissue (Table 1). The lack of any detectable virus correlated with the lack of neurological signs and the lack of virus in plasma at this later timepoint. Thus, early viremia in young animals did not lead to a more widespread infection and did not lead to infection in the brain.

### Innate immune response to LACV infection in different aged macaques

Because we detected differences in viral RNA levels in the plasma between animals, we next asked whether this was associated with specific immune responses. We first analyzed proinflammatory cytokines in the plasma, using a multiplex bead array (Sup. Figure 1). Of the 26 cytokines analyzed, 15 were below the limits of the standard curve for detection. For the detected cytokines (CCL2, CCL5, CCL11, CCL22, CXCL9, IL2, IL-12, IL-1RA, FGFB, HGF, MIF), protein levels were not altered over the time course of infection, except for one adult animal (AL-3) which had increased expression of several cytokines including CCL2 and IL-12 at later stages of infection (Sup. Figure 1 C, F). No consistent and substantial differences were observed with any of the cytokines between the age groups indicating that LACV infection of cynomolgus macaques did not substantially impact plasma cytokine levels in an age-dependent manner.

**Fig. 3:**
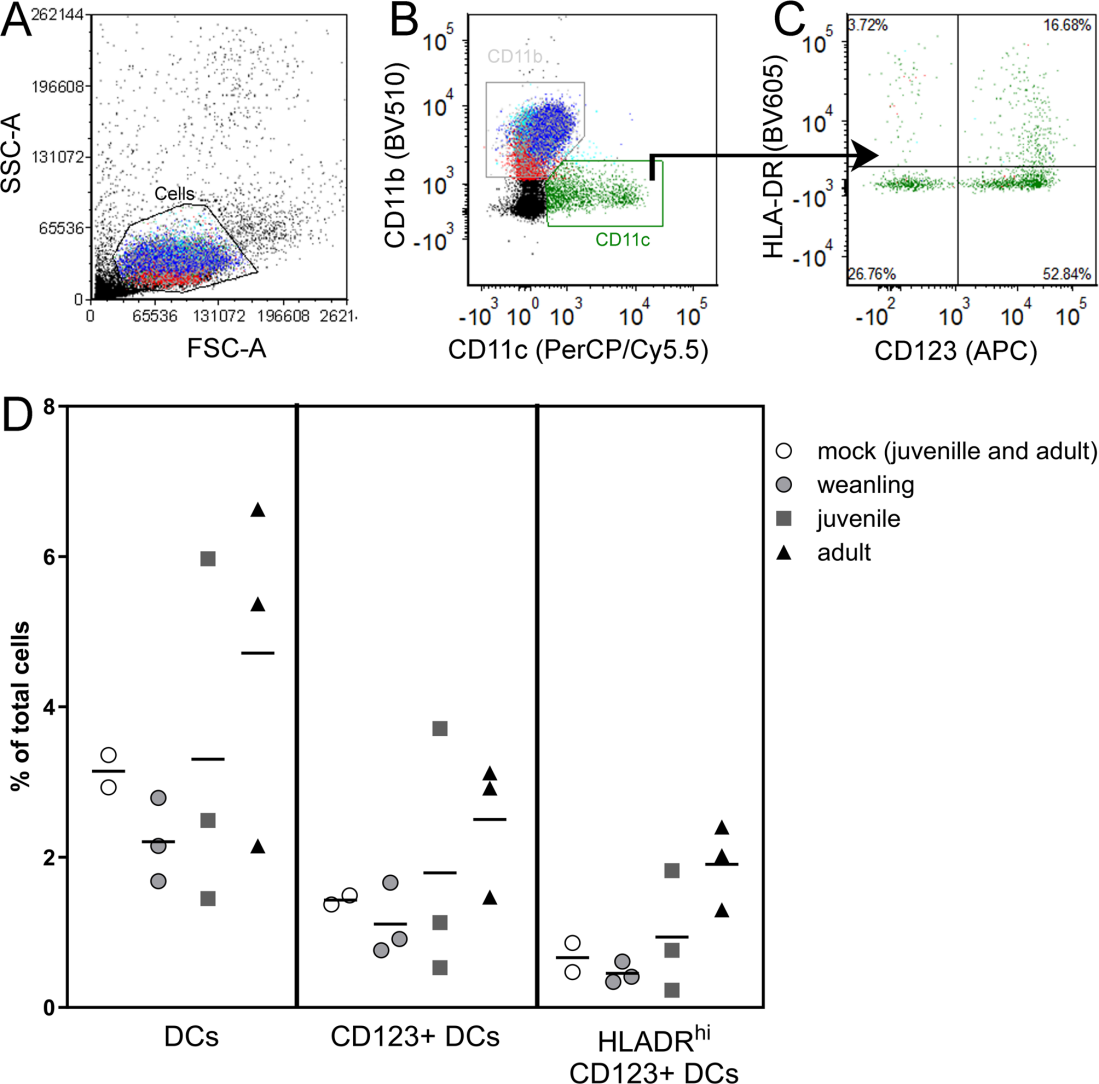
There are minimal age-dependent differences in the dendritic cell response to LACV. 3 dpi flow cytometric analysis of PBMCs from LACV-infected weanling, juvenile and adult cynomolgus macaques was performed to identify CD11c^+^ dendritic cells (**A**,**B**), CD11c^+^ CD123^+^ plasmacytoid dendritic cells and CD11C^+^ CD123^+^ HLADR^hi^ activated plasmacytoid dendritic cells (**C**). Quantifications of the proportions of each cell type as percent of total cells is in shown in (**D**). A Wilcoxon matched-pairs signed rank test was used to compare the proportions of cells in each age group.

**Fig. 4:**
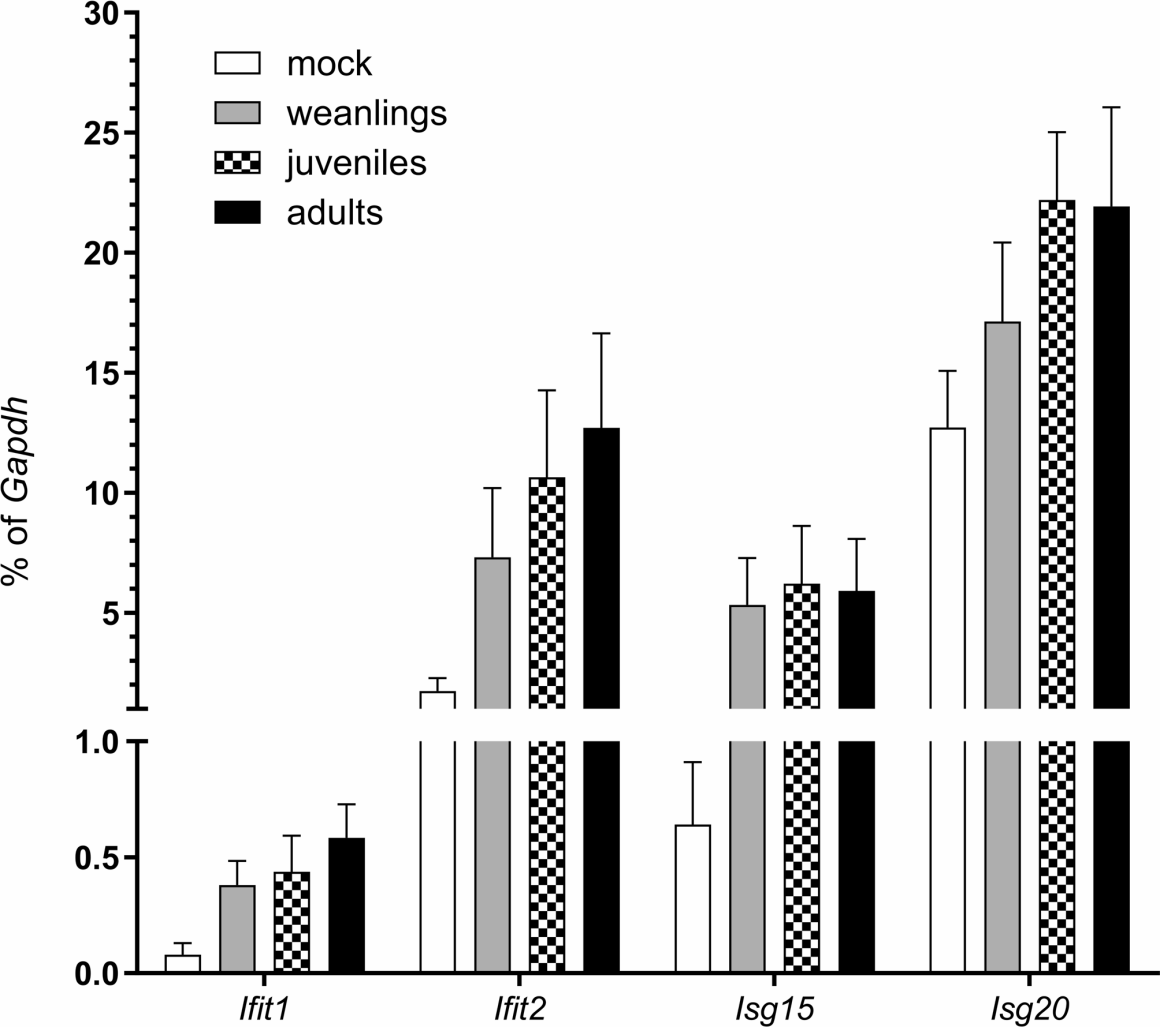
ISG responses in PBMCs from mock and LACV infected cynomolgus macaques were similar regardless of age. ISG transcript expression from PBMCs at 3 dpi was compared between LACV infected weanling, juvenile and adults NHPs and a mock infected juvenile and adult NHP. Data are presented as target transcript expression as a percent of the house-keeping gene *Gapdh*.

We next analyzed whether age influenced the DC response to LACV infection. We analyzed PBMCs at 3 dpi for CD11c^+^ cells as well as specifically looking at plasmacytoid CD123^+^ DCs and HLADR^hi^ CD123^+^ activated plasmacytoid DCs (Fig. 3A-C). A lower percentage of DCs were found in PBMCs from weanling animals compared to adults (Fig. 3D), similar to that observed in naïve animals (Fig. 1F). Additionally, there were lower percentages of CD123^+^ plasmacytoid DCs and activated plasmacytoid DCs in weanling animals compared to adults, although these differences were not significant (Fig. 3C, D). Similarly, analysis of ISG responses at 3 dpi, showed no significant difference in mRNA expression of *Ifit1*, *Ifit2*, *Isg15* or *Isg20* between age groups, although for all except *Isg15*, expression trended higher in older animals (Fig. 4). Thus, there was no outstanding age-related ISG response in PBMCs 3 days post LACV infection, despite some differences in DC populations and a modest increase in ISG response with age.

### Adaptive immune responses to LACV in different age macaques

We next determined whether there were age-related differences in the adaptive immune response to LACV. Analysis of neutralizing antibodies in the plasma of infected macaques at the end of the study (23–28 dpi) demonstrated that all infected animals had neutralizing antibodies to LACV (Table 1). There was no clear correlation between neutralizing antibody titers and early detectable virus levels, however weanling and juvenile animals tended to have higher neutralizing titers than adults (Table 1). Taken collectively, the humoral immune response to LACV infection in macaques was only minimally age dependent.

**Fig. 5:**
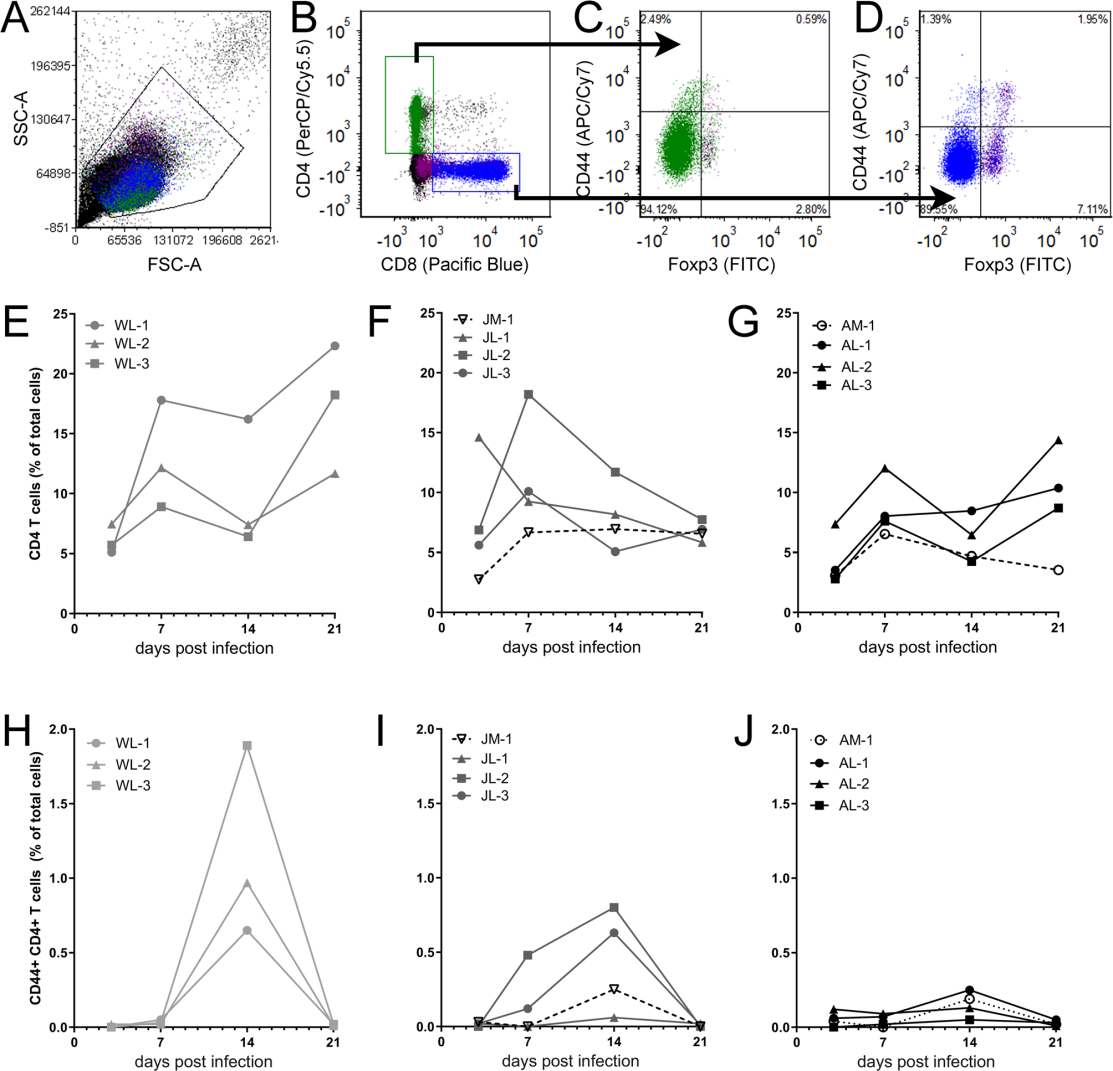
Activated CD4^+^ CD44^+^ T cells are proportionally increased in LACV-infected young NHP. PBMCs isolated from mock and LACV-infected weanling, juvenile and adult cynomolgus macaques were analyzed by flow cytometry for expression of T cell markers (**A**,**B**) including CD44 which is a marker of T cell activation (**C**,**D**). The proportion of CD4^+^ T cells was determined in LACV-infected weanling (**E**), and mock and LACV-infected juvenile (**F**) and adult (**G**) animals. Activated CD4^+^ CD44^+^ T cells were determined in the same animals (**H-J**). All data are presented as the proportion of T cells within total PBMCs.

Analysis of T cell responses to LACV infection was measured by flow cytometry quantification of PBMCs. T cells were gated by CD4 or CD8 expression and then analyzed for activation using CD44 (Fig. 5A-D). The proportion of CD8^+^ T cells varied substantially between different animals throughout infection (Supplemental Fig. 2A-C). Activated CD8^+^ T cells generally peaked at 14 dpi in weanling, juvenile and adult animals (Supplemental Fig. 2D-F), however these data are difficult to interpret because mock animals also had a spurious increase at the same timepoint.

In contrast, CD4^+^ T cell responses did show age-related differences. In weanling animals, the proportion of CD4^+^ T cells amongst PBMCs largely increased throughout infection (Fig. 5E) while, CD4^+^ T cell proportions were largely flat or trended down in juvenile and adults (Fig. 5F-G). A corresponding, large increase was observed in the activated CD4^+^ T cell proportion in weanling animals (Fig. 5H) at 14 dpi. Juvenile macaques had a smaller activated CD4^+ ^T cells response in 2 of 3 animals at the same time point (Fig. 5I) but the response was largely not induced in adult macaques (Fig. 5J). Thus, weanling animals appear to generate a more robust CD4^+^ T cell response to LACV infection than older animals.

### Pathogenesis and immune response to intranasal LACV inoculation in common marmosets

Because infection in cynomolgus macaque did not result in LACV encephalitis, we examined if the adult common marmoset was potentially susceptible to LACV. To increase the chance of brain infection, we inoculated via the intranasal (i.n.) route, as this route bypasses adult resistance in the mouse model^20,21^. Additionally, we used a high dose of 10^6^ PFU of virus per marmoset. However, no animals developed clinical symptoms over a 23-day period. Analysis of immune cell activation in blood was limited due to a lack of reagents and was mostly unchanged by infection (Sup. Figure 3 A-B). However, there was an increase in activated CD4^+^ cells between 7 and 14 dpi (Sup Fig. 3C) and all animals seroconverted (Table 2), suggesting that there was sufficient virus infection to elicit a CD4^+^ T cell and neutralizing antibody response. Nasal swabs taken on 3, 7, 14 dpi were largely negative for viral RNA except for a faint positive for CM-1 on 7 dpi.

**Table 2:** Common marmosets.

Tattoo^a^	Months	Sex	Viral RNA in nasal swabs^b^			Neutralizing antibody^c^	Clinical signs	IHC
			3 dpi	7 dpi	14 dpi	21 dpi		
CM-1	38	F	-	+	-	512	-	Nasal mucosa infection and pathology (23 dpi)
CM-2	36	F	-	-	-	2048	-	-
CM-3	81	F	-	-	-	2048	-	-

^a^: CM = common marmoset. ^b^: ++: 3 of 3 wells positive (detects down to 10 PFU); +: 2 wells positive; - : 0–1 well positive. ^c^: dilution of plasma that neutralized ≥ 50% of virus plaque formation of a known virus stock. -indicates no clinical signs and no histological or immunohistological findings.

**Fig. 6:**
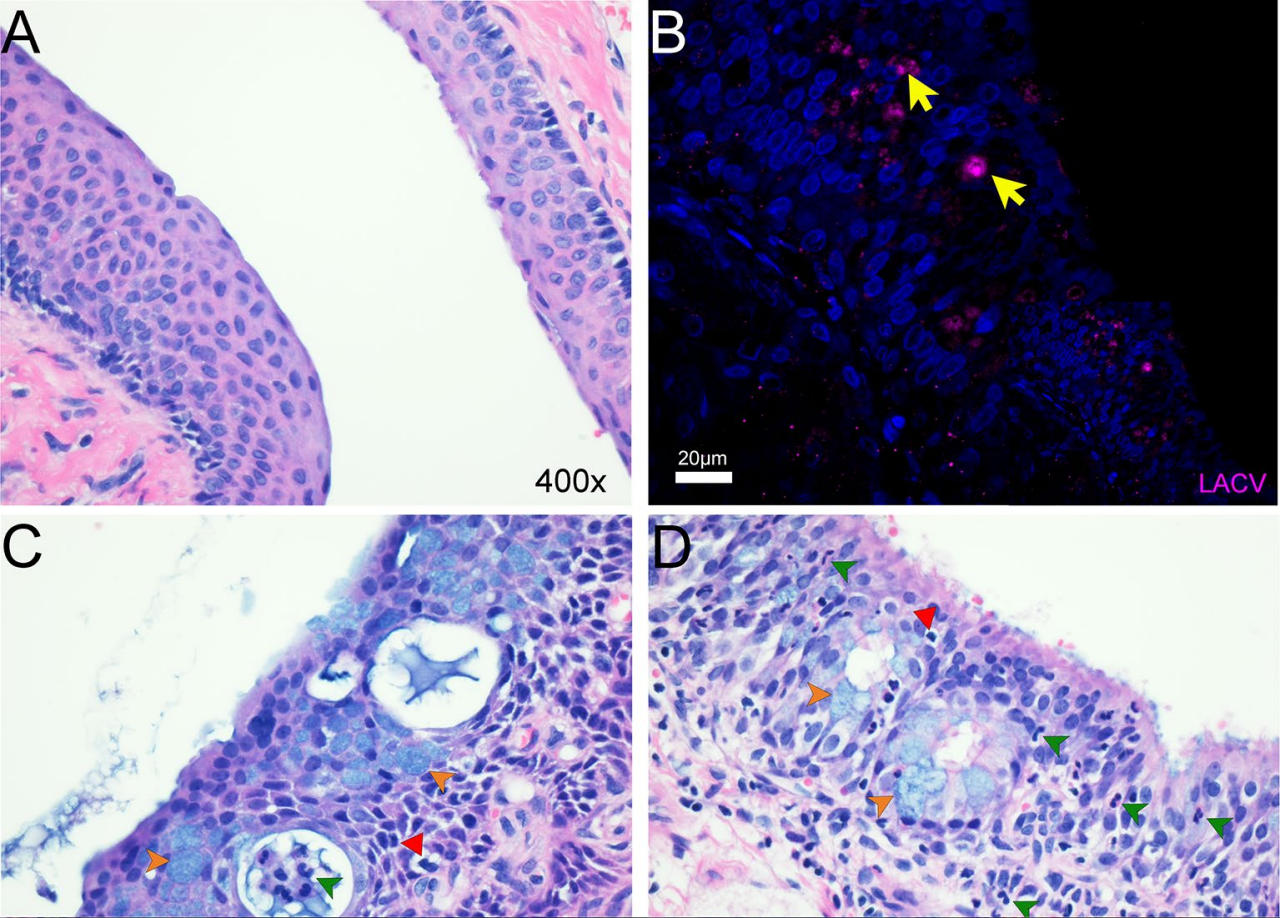
Intranasally inoculation of LACV induces pathology in the nasal epithelium of the common marmoset. Nasal turbinate tissue from marmoset MA250 was stained with H&E (**A**,**C**,**D**) and fluorescently immunolabled with a polyclonal antibody raised against LACV (**B**). Areas of infection were infrequent and delimited with most tissue being normal in appearance (**A**). In areas of infection pathology (**B**-**D**), LACV protein was evident by immunolabeling (**B**, yellow arrows) which was at times coincident with vacuoles (lower arrow in **B** and **C**,**D**) or adjacent to vacuoles (upper arrow in **B**). Hypertrophic goblet cells were also present in areas of vacuolization (**C**,**D**, orange arrowheads) indicating increased mucus production in the area. Vacuoles were commonly found near degenerating cells (**C**,**D**, red arrowheads) and infiltrating immune cells (**C**,**D**, green arrowheads). Edema was commonly observed in the basal cell layer near areas of infection (**D**, *). Images of H&E staining (**A**,**C**,**D**) were all taken at 400x. The scale bar in (**B**) = 20 μm.

Immunohistochemical and histological analysis of brain tissue from all animals was largely unremarkable, with no staining for virus. However, the nasal mucosa of CM-1 at 23 dpi had delimited areas of infection both in cells (Fig. 6B) and in vacuoles formed within the epithelium (Fig. 6B and C). Other areas, particularly in the anterior nasal epithelium, were normal appearing (Fig. 6A). In infected areas, vacuoles contained either infiltrating immune cells or acellular filamentous material (Fig. 6C and D). Hypertrophic goblet cells were present throughout the infected nasal epithelium, most notably around vacuoles, indicative of increased mucus production. Areas of infection were also associated with neutrophil infiltration and edema in the basal cell and mucous gland layers (Fig. 6D) Thus, our findings indicate a prolonged (out to 23 dpi) LACV infection in the nasal mucosa of at least one common marmoset that is associated with tissue inflammation and immune cell recruitment but that had not led to infection of the brain.

## Discussion

In the current study, we examined how age affected the immune response to LACV infection in cynomolgus macaques. We examined three animals younger than 1 ½ years, three animals between 1 1/2 − 2 years and three adult animals that were 6 years or older. Naïve weanling macaques had fewer DCs in their blood than juvenile and adult animals and generated weaker ISG and chemokine responses to both poly I:C and LACV stimulation as compared to older animals (Figs. 1 and 2). This suggests the initial IFN response in weanling animals is not as robust as older animals which correlates with our findings in weanling mice^11^. However, unlike infected weanling mice, weanling macaques eventually mount an ISG response comparable to older animals by 3 dpi (Fig. 4) suggesting they still may sufficiently limit hematogenous virus to prevent invasion of the brain^21^. This finding may explain why cynomolgus macaques, regardless of age, are resistant to LACV-induced encephalitis and show no evidence of brain pathology.

Susceptibility to disease could also involve species-specific anatomical differences, and/or differences in viral tropism. While age-specific studies have not been performed, there are significant differences in drug penetration across the blood brain barrier between mouse and cynomolgus macaques^22^, with macaques being more exclusive. This could suggest replicating virus is less able to access the brain in these species due to a more robust barrier and could help explain our findings. In mice, when LACV is administered via intranasally or intracerebrally to by-pass the blood brain barrier, it replicates to very high levels quickly within neurons and is uniformly lethal^11,20,21^. This contrasts with a recent study where a high dose of LACV was administered intrathalamically to rhesus macaques, but the neuronal infection was largely controlled with 14 days and no animals developed overt neurological symptoms^16^. In this study they determined there was a robust innate and adaptive response within the brain and concluded this was sufficient to clear virus. They also demonstrated morphological and transcription changes in neurophysiologic processes that could represent mechanisms to limit neuron loss. Thus, it is possible that neurons in mice are more susceptible to both LACV infection and LACV-induced cell death than neurons in macaques. Alternatively, macaques may have stronger innate response in glia cells that controls virus infection and limits virus replication within the CNS.

Interestingly, despite a robust ISG response in infected weanling macaque PBMCs at 3 dpi (Fig. 4), viral RNA persisted in the plasma in two out of three animals out to 7 dpi which was longer than in any infected juvenile or adult (Table 1). This suggests that in weanling animals the IFN response may not be entirely sufficient to completely control virus replication. Such a deficiency might be attributable to a faster resolution of the innate response in younger animals. A similar phenomenon was observed in children infected with SARS-CoV-2 where an initially robust DC and ISG response quickly resolved in favor of elevated expression of genes associated with B cell activation^23^. Additionally, the shortened innate response in children correlated with a larger lymphocytic response throughout infection as compared to adults. This is like our findings where weanling macaques generate a robust adaptive response in the form of neutralizing antibody (Table 1) and had higher proportions of activated CD4^+^ T cells compared to adults (Fig. 5). Thus, because weanlings generally have higher viral RNA in plasma compared to older animals, they may need to mount a more robust adaptive response. Considering our previous work demonstrating the adaptive response is critical to disease prevention in adult mice^14^, the current findings may further explain disease resistance in weanling macaques.

The increased CD4^+^ T cell response in weanling animals may simply be a response to higher viral loads at early timepoints (e.g. 3 or 7 dpi) driving CD4^+^ T cell activation^24^. Nonhuman primates have been shown to elicit strong CD4^+^ T cell responses to RNA virus infection in the absence of a CD8^+^ response^25^. This CD4^+^ response also correlated with a decrease in viremia. However, in this study, there is not a clear correlation between detection of viral RNA (Table 1) and activated CD4^+^ T cells (Fig. 5H-I), as WL-2, JL-2 and JL-3 had robust CD4^+^ T cell activation, but no detectable viral RNA. This incongruence may simply be because viral RNA can be difficult to detect in plasma and may have been below the limit of detection. Studies of virus levels have shown the lower sensitivity of detecting virus in plasma rather than tissues, suggesting plasma virus titers are more likely to have false negatives rather than false positives^26^. It is also possible that LACV is replicating in tissue, such as muscle, which is resistant to virus-induced death and has only limited viral release^17^. This persistent replication could continue to drive CD4^+^ T cell activation resulting in the elevated proportions observed at 14 dpi without detectable viremia. Thus, the heightened CD4^+^ T cell activation in WL-2, JL-2 and JL-3 could be due to virus that was just below the limit of detection in the younger animals.

It was also surprising to observe that adult macaques had very modest activated CD4^+^ T cell responses throughout infection (Fig. 5J). This lack of CD4^+^ T cell expansion in adults may also be related to the modestly lower neutralizing antibody titers observed in adult animals relative to younger animals (Table 1) as CD4^+^ T cells are critical to induce a robust antibody response to viral infections^27^. It is possible that a more robust IFN response in adults is effectively clearing virus early in infection leading to these weaker CD4 and neutralizing antibody responses. Our findings that poly I: C and LACV stimulation of PBMCs elicit a larger ISG response in adults (Fig. 2) support this interpretation.

Because macaques did not develop encephalitis, we attempted to develop a NHP LACV encephalitis model by circumventing the immune responses in the susceptible common marmoset by infecting i.n. with LACV. All animals were asymptomatic out to 23 dpi, however, we did observe delimited infection and inflammation in the nasal mucosa of one marmoset at the end point. This finding was surprising because it indicated persistent LACV infection in the nasal mucosa which has not been reported. This finding could suggest LACV tropism within the common marmoset is altered relative to other species. Previous work in mice demonstrates i.n. administration of LACV results in infection limited to olfactory sensory neurons within the nasal tubinates^21^. However, infection of sensory neurons was not immediately obvious in the marmoset nasal mucosa by correlative H&E and immunohistochemistry for virus (Fig. 6). It would be interesting to expand this analysis when the molecular tools to identify neurons in this region have been developed^19^.

Persistent LACV infection in the nasal mucosa theoretically could have facilitated our original hypothesis of retrograde trafficking of virus along infected olfactory sensory nerves to invade the brain. Alternatively, the substantial vascularity of the nasal mucosa could have allowed for viral hematogenous spread to the brain. Both invasion routes have been verified in murine models^9,21^. However, encephalitis did not occur. One possible reason for the lack of encephalitis is that the immune system controlled virus spread, which correlates with the observation that sites of LACV infection in the nasal mucosa were delimited. This idea is supported by our finding that all animals had measurable neutralizing antibody responses (Table 2). Furthermore, these infection sites were associated with infiltrating immune cells (Fig. 6), and we demonstrated activated CD4^+^ T cells responses were present in all animals (Sup Fig. 3). Thus, the persistent infection could have been sufficiently controlled to prevent infection of olfactory sensory neurons or virus release in the blood. Further studies will be required to validate this possibility. Collectively our findings suggest marmosets could be an interesting model to study the mucosal immune response to LACV.

An alternative possibility for the absence of encephalitis in marmosets is that infection in the nasal mucosa is not substantial enough to allow for spread to the rest of the body. Supporting this idea, we only found evidence of infection in delimited and infrequent areas of the nasal mucosa (Fig. 6). The nose is not the natural route of LACV infection, thus it possible that other tissues may be more amenable to propagation of infectious virus. We have previously demonstrated that the panniculus carnosus muscle in mice is the initial site of LACV infection via the s.c. route^17^. This tissue may also be susceptible to infection in marmosets. Furthermore, it would be interesting to examine age dependent susceptibility to LACV encephalitis in marmosets. It is possible the immune response to LACV in younger animals would be less robust than in adults as have been demonstrated in mice^11,14^. Thus, future studies utilizing s.c. infection in young marmosets may result in LACV encephalitis.

Collectively our immunological findings suggest a potentially weaker innate immune response in weanling animals in the first hours-to-days that is less effective at clearing virus infection than the early response in juvenile or adult animals. The later, activated CD4^+^ T cells response in weanlings is stronger than those measured in older animals suggesting it may be compensating for the initial weaker innate response. Despite these age-dependent differences in the immune response, all animals sufficiently controlled infection to prevent encephalitic disease. These immune responses are likely similar to those in human children, as the vast majority of human infections are asymptomatic^5^. Thus, due to the limited number of infected animals in this study, the outbred nature of cynomolgus macaques^28^, and the similarity of their immune response to humans^29^, it is not surprising we did not observe disease. It is possible that a certain percentage of weanling macaques would be susceptible to LACV, however NHP research on that scale is not feasible. Additionally, although our further attempt to develop a NHP model of LACV encephalitis in 3 common marmosets by i.n. inoculation did not result in disease, future studies that use an alternate route of infection in marmosets may be more successful.

## Methods

### Animals, ethics and clinical analysis

All infectious work with NHPs in this study was conducted with approval and oversight from the Rocky Mountain Laboratories (RML) Institutional Biosafety Committee and the RML Animal Care and Use Committee (ACUC). Studies were conducted in an AAALACi accredited facility following the guidelines and basic principles in the Guide for the Care and Use of Laboratory Animals, the Animal Welfare Act and the U.S. Public Health Service Policy on Humane Care and Use of Laboratory Animals. The study design was constructed with consideration for the Animal Research: Reporting of In Vivo Experiments (ARRIVE) guidelines, but with the knowledge that a limited number of NHPs were available. All methods are reported in accordance with these guidelines and all relevant NHP information is reported in Tables 1 and 2.

Mauritius origin cynomolgus macaques obtained from NIH colonies, were individually housed in adjoining primate cages that enabled social interactions. Marmosets obtained from NIH colonies were socially housed to mimic natural social structure. Commercially available nonhuman primate diets were provided twice daily and supplemented with treats, vegetables, or fruit daily. Water was available *ad libitum*. All animals were housed under controlled conditions of temperature, humidity, and light (12 h light:12 h dark cycles). Environmental enrichment consisted of a variety of manipulanda, commercial toys and human interactions. All procedures were conducted on anesthetized animals under the supervision of veterinary staff. Anesthetized was delivered for inoculation, blood draws and euthanasia at the indicated time points to cynomolgus macaques via intramuscular (i.m.) injection of ketamine (8-12 mg/kg) and to marmosets via i.m. injection of ketamine (20 m/kg)-xylazine (2 mg/kg) or isoflurane inhalation to effect per ACUC guidelines. Analgesics or other treatment methods could interfere with infection, the immune response to infection or the development of neurologic symptoms and were not used in this study^30–32^.

For all animal studies, endpoint criteria were specified by RML ACUC-approved parameters to determine when animals were to be humanely euthanized. This unbiased approach removed the need for experimental blinding or randomization. Clinical scoring was performed twice daily and was based on the following criteria: general appearance, skin and coat appearance, discharge, respiration, neurological signs, food intake, feces and urine output and activity. A cumulative minimum clinical score was established to assist with early timepoint humane euthanasia decisions. In anticipation of viral infection of the brain, any sign of neurological disease was a clinical endpoint, although this was never reached by any study animal. Other nonviral pathologies were also considered. In the case of marmosets CM1 and CM2, hepatic issues at the 23 dpi timepoint were grounds for euthanasia. Additionally, lethargy and lack of grooming by marmoset CM3 at the same timepoint triggered euthanasia criteria. Although no cynomolgus macaques met endpoint criteria, WL-1, JL-1 and AL-2 were euthanized at 23 dpi such that histopathology could be performed on tissues. This decision was made, because WL-1 and JL-1 had demonstrated viral RNA in plasma at earlier timepoints (Table 1) and AL-2 was included as a comparison for the adult group. Euthanasia was performed under anesthesia (described above) by intravenous (i.v.) or intracardiac administration of Euthasol^®^ euthanasia solution (1mL/5kg, Virbac).

### Virus stocks, NHP infections and whole blood collections

A 1978 human isolate of LACV was used for all experiments and has previously been described^9^. Infectious virus had not been passaged more than twice in vitro. LACV stocks were generated and titered by plaque assay as previously described^33^. Cynomolgus macaques were infected subcutaneously (s.c.) along the dorsal thorax with 500µL of straight stock inoculum at a dose of 10^7^ plaque-forming units (PFU)/mL. Common marmosets were infected intranasally (i.n.) with 500µL of stock inoculum diluted 1:10 in sterile, pharmaceutical grade PBS at a dose of 10^6^ PFU/mL. 250µL of inoculum was applied to each naris. At indicated timepoints before and after LACV infection, whole blood was collected via i.v. puncture into EDTA-treated Vacutainer^®^ tubes (BD). Collected volumes did not exceed 10% of circulation blood volume in a two-week period for either species.

### In vitro PBMC stimulation

Prior to LACV infection, EDTA whole blood from weanling, juvenile or adult cynomolgus macaques or adult common marmoset was collected. Most red blood cells (RBCs) were removed from each sample by diluting 1:2 into PBS containing 1% Dextran T-500, mixing and incubating each for 30 min at 37 °C. The partially clarified top layer containing PBMCs was collected, diluted 1:2 in PBS and spun for 5 min at 500 g. The pellet was resuspended in 3mL of ACK lysis buffer and incubated 5 min to remove any remaining RBCs. Lysis was halted by diluting the lysis buffer 1:3 with PBS and centrifuging for 5 min at 500 g. Pelleted cells were resuspended in warm RPMI-1640 supplemented with 10% fetal calf serum and 5% Penicillin-Streptomycin at a concentration of 2*10^6^ cells/mL. 500µL of PBMCs were added to each well of a 24 well plate. Treatment media for mock, high molecular weight (HMW) poly I: C (Invivogen) and virus were made up separately at a 2x concentration and 500µL was applied to each well. Mock media contained volume-matched Vero supernatant relative to the LACV media. HMW Poly I: C was diluted into 0.5 mg/mL Lyovec transfection reagent (Invivogen) to a concentration of 200 µg/mL and then diluted 100-fold into culture media such that the final concentration applied to PBMCs was 1 µg/mL. The final multiplicity of infection of LACV-contained media when applied to PBMCs was 0.01. PBMCs were incubated at 37 °C for 16 h after which cells were harvest into Eppendorf tubes and centrifuged for 5 min at 500 g. Cells were washed with PBS and repelleted at 500 g. Cells were then lysed with ZR RNA buffer (Zymo) for later quantitative real-time PCR analysis.

### Flow cytometry

Following infection, PBMCs were isolated from EDTA whole blood of weanling, juvenile or adult NHPs as described above at the indicated time points. PBMCs were suspended in PBS with 0.05% BSA on ice at 10 × 10^6^ cells/mL following counting with a hemocytometer. 100µL of each sample was added to each required well of a 96 well plate to be immunolabeled. PBMCs were then incubated with TruStain FcX™ (Biolegend) Fc receptor blocking solution for 30 min and the immunolabeled with direct conjugate antibodies (Table 3). Controls included unlabeled cells, single antibody labeled cells and all antibodies minus-one labeled cells. PBMCs were then washed 3x with PBS, fixed with 2% paraformaldehyde for 30 min and washed again 3x with PBS and analyzed using a BD LSRII (BD Biosciences). Flow cytometric analysis was performed using FCS Express Research Edition version 5 (Denovo software). Gating strategies for specific cell types are shown in the respective figures.

**Table 3:** Primary conjugate antibodies used for flow cytometry.

Species application^a^	Antigen	Fluorochrome	Clone	Source
MF	CD11b	BV510	ICR 544	BioLegend
MF	CD11c	APC	S-HCL-3	BioLegend
CF	CD11c	BV421	3.9	BioLegend
MF	CD123	PerCP/Cy5.5	6H6	BioLegend
MF and CF	CD14	AF700	M5E2	BD Biosciences
MF and CF	CD16	APC/Cy7	3G8	BD Biosciences
MF and CF	CD4	PerCP/Cy5.5	L200	BD Biosciences
MF and CF	CD44	APC/Cy7	IM7	BioLegend
MF	CD8	PB	SK1	BioLegend
MF	Foxp3	FITC	150D	BioLegend
MF and CF	HLA-DR	BV605	L243	BD Biosciences

^a^: M.F.=*Macaca fascicularis* and C.F.=*Callithrix jacchus*.

### RNA isolation from plasma and PBMCs and real-time PCR analysis

For viral RNA isolation from plasma at the indicated time points, EDTA whole blood was centrifuged at 2000 g for 10 min and the upper plasma fraction collected. RNA was extracted from 100µL of plasma. For viral RNA isolation from nasal swabs, the tip of the swab was incubated in 0.5mL of Viral RNA Buffer (Zymo) for 30 min with mixing. Subsequent RNA isolation from both plasma and nasal samples was performed using the Zymo ZR Viral RNA Isolation Kit (Zymo) per the manufacturers’ column-based protocol. Isolated RNA was eluted from the columns using 15µL of nuclease-free H_2_0. For RNA from PBMCs, cells were isolated from EDTA whole blood as described above. PBCM RNA was isolated using the Quick-RNA™ Miniprep Kit (Zymo) per the manufacturers’ column-based protocol. Isolated RNA was eluted using 35mL of nuclease-free H_2_0. Generation of cDNA from plasma and cell RNA samples and analysis of host transcripts was performed as previously described^34^. For host genes, transcript expression was plotted relative to *Gapdh* expression. Analysis of viral RNA from plasma was done by comparing Ct values from plasma samples to standards established by spiking naïve species matched NHP plasma with null, 10^1^, 10^2^, and 10^3^ PFU. Samples were run in three triplicate groups and 6–9 wells with Ct values consistently near or above the 10^1^ standard were considered positive, 2–5 wells were considered weak positive and 0–1 wells were negative (Table 1). Analysis of viral RNA from nasal swabs were similar, however, the analysis was only performed in triplicate with Ct values of all 3 wells consistently near or above the 10^1^ standard being considered positive, 2 wells considered weak positive and 0–1 wells negative (Table 2). Primers for all transcripts are shown in Table 4.

**Table 4:** Primers used for quantitative real-time PCR analysis.

Gene name	Forward primer 5’-3’	Reverse primer5’-3’
LACV	ATTCTACCCGCTGACCATTG	GTGAGAGTGCCATAGCGTTG
*Ifit1*	GCTTTCAAATCCCTTCCGCT	GCCTTGGCCCGTTCATAATT
*Ifit2*	CCGAACAGCTGAGAATTGCA	CCCTCCATCAAGTTCCAGGT
*IL-1b*	ACGATGCACCTGTACGATCA	GGAGGTGGAGAGCTTTCAGT
*IL-6*	CCCTGACCCAACCACAAATG	AAGCTGCGCAGAATGAGATG
*Isg15*	TTGCCAGTACAGGAGCTTGT	GGGACACCTGGAATTCGTTG
*Isg20*	CGATTACAGAACCCGGGTCA	TTCAGGAGCTGCAGGATCTC
*Oas1*	CGTGTTTCCGCATGCAAATC	CACCTTGGACACACACACAG

### Plasma cytokine analysis

Concentrations of 29 cytokines, chemokine and growth factors were measured in plasma from mock and LACV infected weanling, juvenile and adult cynomolgus macaque at 3, 7, 14 and 21 dpi using the Luminex magnetic bead-based assay (Monkey Cytokine Magnetic 29-Plex Panel (ThermoFisher)). Plasma was prepared as described above. The assay was run according to the manufacturer’s protocol using 50µL of sample. Protein concentrations were measuring using a Bio-Plex 200 system (BioRad).

### Plaque assay and neutralizing antibody assays

Plasma from individual NHPs were collected and isolated as described above and stored at -80 °C. For plaque assays of NHP plasma, C17.2 cells (Sigma-Aldrich) were plated 1 day in advance into 24-well plates to confluency. Plasma samples were diluted serially at 1:10 decreasing concentrations for initial screening and 1:2 for final dilutions in DMEM (Gibco) supplemented with 2% FBS and penicillin/streptomycin and 200 µl of each dilution was applied the Vero cultures in duplicate. Plates were incubated 1 h to allow virus attachment and then each well was overlayed with 0.5 mL of 1.5% carboxymethyl cellulose in MEM (Gibco). Plates were incubated for 5 days and then fixed with 10% formaldehyde to a final concentration ≥ 4% formaldehyde per well for 1 h. Wells were rinsed with water, stained with 0.35% crystal violet and rinsed with water again. Plates were air dried and scanned for plaques.

For neutralization assay, plasma was diluted 1:10 and then subsequent 1:5 in DMEM, 2% FBS, 1% P/S. Samples were then mixed with 10^2^ plaque-forming units (PFU) of LACV in DMEM/FBS/P/S. The entire mixture was incubated for 1 h at 37 °C to achieve neutralization. After neutralization, the mixture was added to confluent Vero cells in a 24-well plate and incubated again for 1 h at 37 °C. After incubation, 1.5% carboxymethylcellulose (CMC) in modified Eagle medium (MEM) was overlaid onto the Vero cells and incubated undisturbed at 37 °C. After 5 days the mixture was fixed by adding 10% formaldehyde to each well and incubated for 1 h at room temperature. After fixation, plates were rinsed with de-ionized water wash and stained with 0.35% crystal violet. Neutralizing titer was determined by the dilution that inhibited 50% viral PFUs compared to a 10^2^ LACV-infected control well.

### Histology and immunohistochemistry

Necropsies and tissue sampling was performed on cynomolgus macaques WL-1, JL-1 and AL-2 (Table 1) and all common marmosets (Table 2) at 23 dpi. Tissues collected included brain, spinal cord, dorsal root ganglia, draining lymph node, gut-associated lymph tissue, spleen, lung, muscle, liver and for common marmosets, nasal turbinates. Tissues were fixed in 10% neutral buffer formalin for a minimum of 7 days and processed for paraffin embedding, sectioning, H&E staining and immunohistochemistry as previously described^35^. H&E sections were evaluated by a blinded, board-certified veterinary pathologist for evidence of virus-associated pathology or degeneration. Immunolabeling for LACV was performed with an inhouse-generated rabbit polyclonal antibody that has previously been described^20^. Immunolabeled images were obtain with a Zeiss 710 (Zeiss) laser scanning microscope with a Plan-Apochromat 63x objective (NA of 1.40). Images of H&E-stained tissues were collected on a Nikon Eclipse 55i microscope (Nikon) with a Plan Fluor 40x objective (NA of 0.75) and a DS-Fi3 digital camera at 400x magnification.

### Statistical analysis

All statistical analysis was performed with GraphPad Prism version 10.2.0 (GraphPad Software). Relevant tests are described in the associated figure legend. The authors acknowledge that all experiments have a small sample size due to the limitations of NHP research. It is possible that effects may be missed due to this small sample size, disallowing the rejection of the null hypothesis that may be significant with higher numbers of animals. The interpretation of the presented data takes this into account and thus focuses on both statistical differences and consistent trends that do not reach statistical significance.

## Electronic supplementary material

Below is the link to the electronic supplementary material.

Supplementary Material 1

## Data Availability

All data has been made available within the manuscript.
