# Supplementary material for: Age dependent susceptibility and immune responses to La Crosse virus infection in non-human primates
Source: Sci Rep. 2025 May 13;15:16628. doi: 10.1038/s41598-025-01285-8 (PMC12075599; doi:10.1038/s41598-025-01285-8)
Supplement: Supplementary file 1 — Supplementary Material 1 [file 41598_2025_1285_MOESM1_ESM.docx]

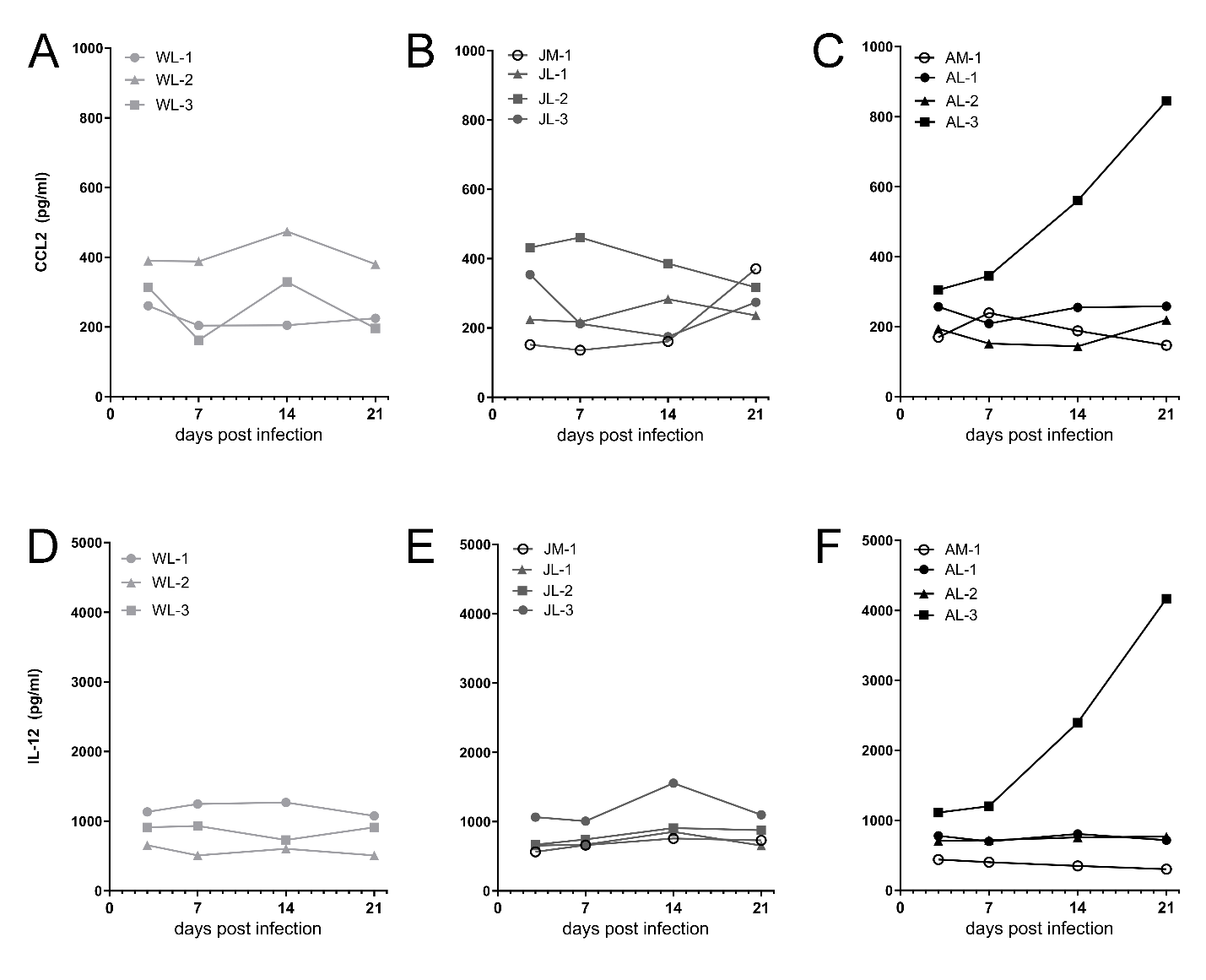
Supplemental Figure 1. Plasma cytokines are similar in LACV-infected cynomolgus macaques regardless of age. Plasma isolated from the blood of mock and LACV-infected weanling, juvenile, and cynomolgus macaques at 3, 7, 14 and 21dpi was Luminex magnetic bead-based assayed for the protein concentration of CCL2 (A-C), and IL-12 (D-F) cytokines. Data are plotted as picograms/mL concentrations.


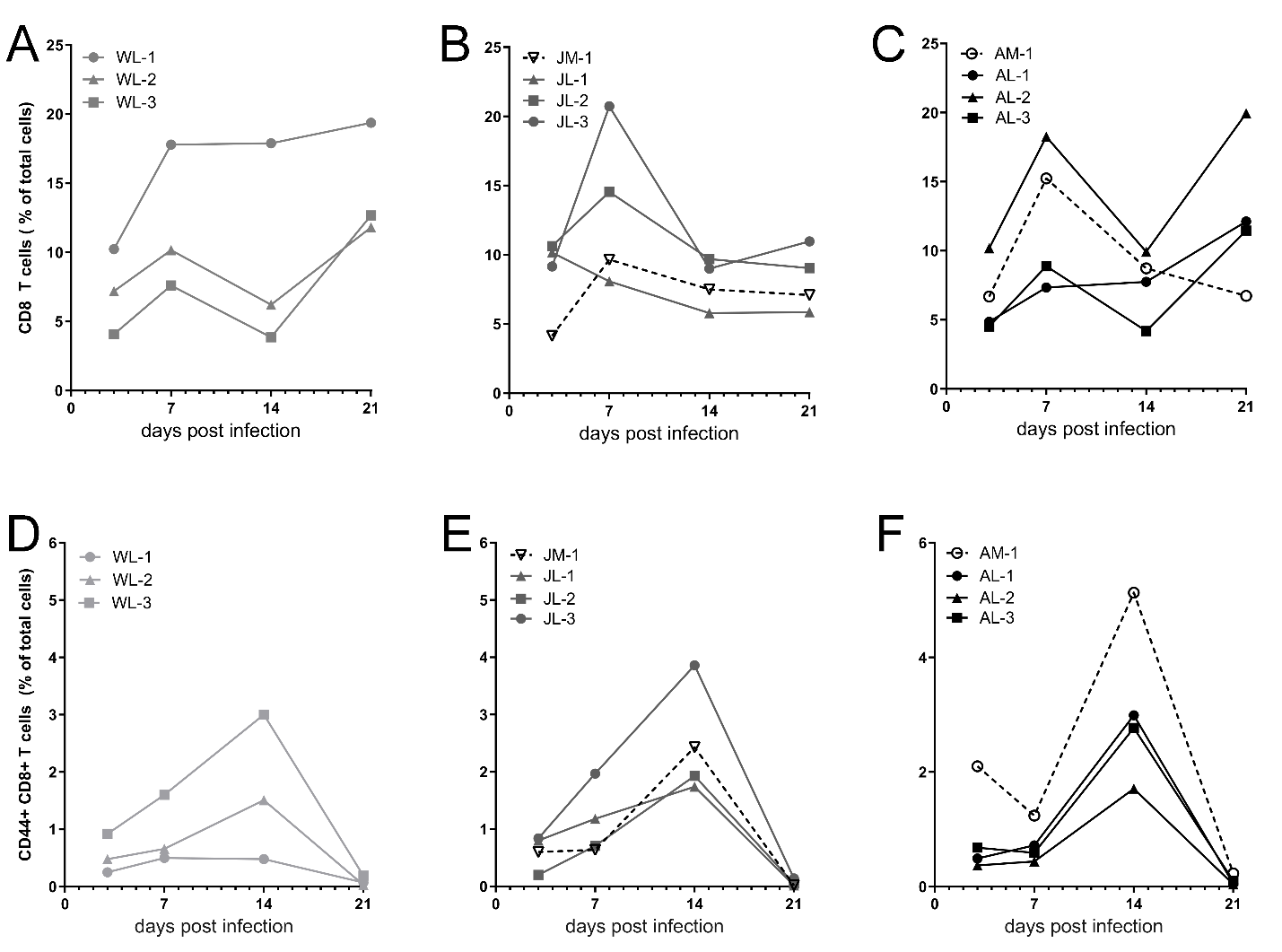
Supplemental Figure 2. CD8^+^ T cell proportions in mock and LACV-infected cynomolgus macaque. The proportion of CD8^+^ T cells was determined in LACV-infected weanling (A), and mock and LACV-infected juvenile (B) and adult (C) animals. Activated CD8^+^ CD44^+^ T cells were determined in the same animals (D-F). All data are presented as the proportion of T cells within total PBMCs.


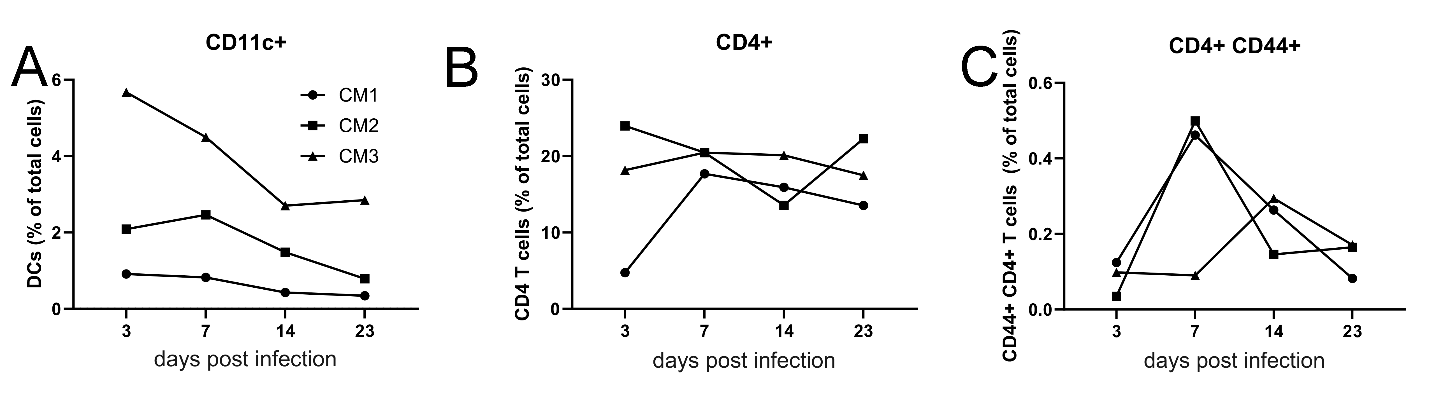
Supplemental Figure 3. Cellular immune response in EDTA whole blood of LACV-infected common marmosets. PBMCs were isolated from the blood of LACV-infected common marmosets at 3, 7, 14 and 21dpi. Using flow cytometry gating strategies for dendritic cells (Figure 3A-B) and CD4^+^ T cells (Figure 5A-C), the proportions of CD11c+ dendritic cells (A), CD4^+^ T cells (B) and activated CD4^+^ CD44^+^ T cells in whole blood were determined. Data are plotted as the portion of each identified cell type as part of total PBMCs.
